# COVID-19: The Conjunction of Events Leading to the Coronavirus Pandemic and Lessons to Learn for Future Threats

**DOI:** 10.3389/fmed.2020.00223

**Published:** 2020-05-12

**Authors:** Roger Frutos, Marc Lopez Roig, Jordi Serra-Cobo, Christian A. Devaux

**Affiliations:** ^1^Cirad, UMR 17, Intertryp, Montpellier, France; ^2^IES, UMR 5214 Univ. Montpellier-CNRS, Montpellier, France; ^3^Department of Evolutionary Biology, Ecology and Environmental Sciences, Biodiversity Research Institute, University of Barcelona, Barcelona, Spain; ^4^IHU-Méditerranée Infection and CNRS, Marseille, France

**Keywords:** COVID-19, SARS-CoV-2, dynamic, preparedness, emerging diseases

## Introduction

Originally identified in December 2019 in Wuhan, China, SARS-CoV-2 has become a pandemic owing to a long period of incubation, a high number of asymptomatic cases, and high international mobility. Here we consider the unique conjunction of events that allowed this new coronavirus to emerge and create a pandemic. We urge governments to learn from SARS and COVID-19 and to implement preparedness for pandemics to come.

## An Unpredictable Accident

Following the emergence of SARS-CoV-2 (1, 2) in China, causing COVID-19, the remaining question is whether we could have been ready for it after learning from the SARS epidemic in 2003. It is not possible to predict the emergence of an infectious disease because it is an accidental process, i.e., the occurrence of a very low probability event resulting from the stochastic conjunction of independent low probability events (3). Even if the SARS-CoV-2 outbreak was unpredictable, we should have been able to prevent it because some features are consistent with previous coronaviruses outbreaks.

## Conditions for the Emergence of an Infectious Disease

**Figure 1:**
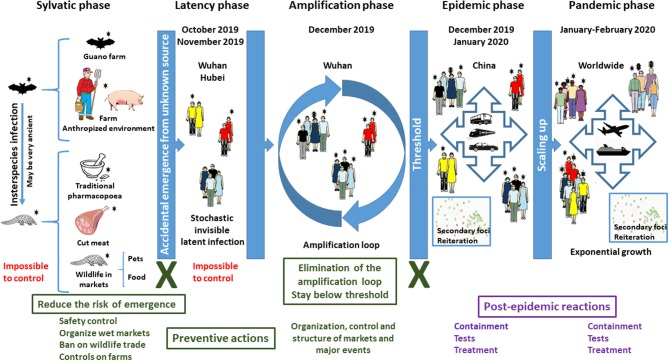
Dynamic of COVID-19. The emergence of an infectious disease is an accidental process that cannot be predicted. An unidentified animal or animal parts contaminated by a virus initially originating from bats, i.e., SARS-CoV-2, was brought into contact with humans in October–November 2019, starting a latent infection. The main drivers of the epidemic and then of the pandemic are human mobility during the incubation phase and the amplification effect of markets, while the extension into a pandemic was due to the dimensions and speed of mobility of goods and people in our global world. However, this did not trigger the outbreak. What favored the epidemic and then the pandemic is the exceptional conjunction in Wuhan of several independent and aggravating events: (i) the occurrence of three major celebrations in a short time, for which the demand of food and natural products was exceptionally high in December 2019; (ii) the resulting movement and storage of large amounts of food including living animals in December 2019; (iii) the very high attendance of markets in December 2019, generating an amplification loop; (iv) very high human mobility for the holidays in January 2020; (v) intensive international mobility of goods and people in January and February 2020; (vi) a long period of silent incubation of SARS-CoV-2. Text in red corresponds to situations where no action can be undertaken. Text and boxes in deep green correspond to situations where preventive actions MUST be implemented to prevent future emergence of SARS-related coronaviruses. Crosses in deep green indicate major transmission steps that can be blocked. Text and boxes in violet correspond to situations where post-event reactions are currently implemented but which cannot prevent a pandemic.

For an infectious disease to emerge, three conditions must be fulfilled. One is of biological nature: the pathogen causing the outbreak must be compatible with humans, i.e., must be able to infect and reproduce in humans (Condition 1). The other two conditions are anthropogenic. First, there must be contact between humans and the pathogen reservoir (condition 2), and, secondly, a human-to-human urban cycle must be possible (condition 3). COVID-19 exemplifies all three conditions (Figure 1), but this is true for all zoonoses.

## Closely Related Viruses

SARS-CoV, which caused SARS in 2003, and SARS-CoV-2, which is responsible for COVID-19, are very closely related Sarbecoviruses. Furthermore, SARS-CoV-2 is closely related (96% similarity) to the Sarbecovirus MN996532_raTG13 from the Chinese horseshoe bat *Rhinolophus affinis* (4). Although 3,200 CoVs circulate in bats (5), it is worth noting that the SARS and COVID-19 pandemics were caused by two very closely related Sarbecoviruses found in Chinese bats. This suggests a specific ability of these Sarbecoviruses to affect humans (condition 1). However, there is also a specific societal environment fulfilling conditions 2 and 3 that led to the COVID-19 epidemic and pandemic.

## The “Planetary Alignment” That Triggered COVID-19

The emergence of COVID-19 is the result of an exceptional “planetary alignment,” a specific coincidence of unrelated natural and societal traits (Figure 1). This leads to condition 2, contact. Although it cannot be excluded, there is no evidence of direct coronavirus infection of humans from bats (6, 7). Civets and dromedaries were intermediate species for SARS and the unrelated MERS coronavirus disease in the Middle East, respectively (8, 9). Similarly, an intermediate animal might have been involved in the emergence of COVID-19. SARS-CoV-2 could possibly infect pangolin, cat, civet, cow, buffalo, swine, goat, sheep, and pigeon (10). Pangolin was mentioned as a potential intermediate, but it is not formally established. COVID-19 is officially considered to have emerged at the Huanan seafood wholesale market (HSWM) in Wuhan in December 2019. However, epidemiological data show that early cases of COVID-19 were not related to HSWM and thus that it is not the site of emergence (11–15). Phylogenetic studies suggest that SARS-CoV-2 might have circulated in Wuhan as early as October 2019 and that the virus then spread at low-level from person to person (the latency phase), before being imported to HSWM where it was detected in December 2019 (13–15). The location of the first human infection will most likely remain unknown. Contamination through traditional medicine, pets, or any other contact event between humans and the source of the virus, including the handling of viruses in a laboratory (16), must be considered. The initial contact might also have taken place in farms, since anthropized rural areas offer favorable environments for the transmission of coronaviruses (3). In this latency phase, the infection remained silent, spreading in a stochastic way within the population, with no epidemic identified yet.

Condition 3 was fulfilled when considering the specific societal context of Wuhan at the end of 2019 and the beginning of 2020. To move from the latency phase to the epidemic phase, an amplification process must occur to reach the threshold needed to trigger an epidemic. The outbreak was initially detected in the Jiang'an district, which is home to the environmentally-conscious Baibuting urban community, which holds a traditional folk festival known as Wan Jia Yan or Great Family Feast every year (17, 18). The 20th such event, organized on January 18, 2020, coincided with the very popular Lunar New Year celebration. More than 40,000 families, who prepared about 14,000 traditional dishes, attended Wan Jia Yan in January 2020 (19). Shops and markets registered a huge attendance of people buying fresh food and, thus, imported and stored large amounts of food, including living animals, in preparation for these events. What triggered the epidemic is the simultaneous occurrence of two major celebrations in the same place, bringing many people into contact with the initially infected persons and providing the amplification phase needed. Another key step was mobility. The Chinese New Year is associated with an outbound mass mobilization known as Chun Yun, and Wuhan is both the heart of the Yangtze River Economic Belt and a major national hub in China known as “the gateway of nine provinces.” An estimated 5 million people left Wuhan during Chun Yun in 2020 (20). Furthermore, Wuhan welcomes 1.2 million college students (21), whose mobility during holidays is extremely high. Outbound traveling from Wuhan may explain why Wenzhou, in the neighboring province of Zhejiang, became one of the most severely affected areas (22). At that stage, it was too late to stop the epidemic, and measures could not be anything but post-event reactions (Figure 1). The expansion was driven in secondary foci by people who moved from the initial location of the epidemic. In each of these foci, the same processes of latency, amplification, and epidemic were reiterated with variable delays. This is why SARS-CoV-2 was not stopped despite drastic measures of containment and quarantine. The next step, global dissemination, was only a matter of dissemination due to intensive international mobility and global international trade.

## What Measures Should be Taken?

Drastic countermeasures for containment were implemented worldwide as a response to COVID-19 that strongly and durably impacted both society and economy but did not efficiently stop the pandemic. The impact of the COVID-19 pandemic is unprecedented in our modern civilization. One must go back to the Spanish flu or black plague in the Middle Ages to find similar societal impacts. Society today is globalized, driven by social networks, and connected with information flowing in real time. This leads to over-reactions, with irreversible damage to society. COVID-19 is the first “4.0 pandemic.” Society cannot allow this situation to repeat in the future and must adapt to implement a different action plan, not based on post-event reactions as done today but rather on preventive actions.

Nothing can be done to avoid the circulation of coronaviruses in the wild (sylvatic cycle). However, the animal intermediate does not need to be identified since human activities are responsible for the emergence and propagation of the zoonosis. The focus must be on these human activities because they can be properly organized. The invariables in both the SARS and COVID-19 epidemics are the presence of living wild animals for trade, food, or medicine, the presence of amplifying nodes like markets (wet or not), large social events, and mobile subpopulations. Following the emergence of COVID-19, the Chinese Government put a ban on the trade and consumption of wild animals, just like after the SARS crisis in 2003–2004. However, these practices are deeply anchored in traditions and are very difficult to proscribe. This is not limited to China or Asia, and the consumption of wild animals is traditional in all continents. Banning wet markets had already been recommended after the SARS crisis (16), but it is not possible in reality, and there is a risk of encouraging illegal markets, with loss of control. For example, following an enforced ban on poultry export from Thailand, the avian influenza H5N1 virus spread widely in Cambodia due to illegal trade from Vietnam through middlemen and wet markets (23, 24). It seems more acceptable for governments to replace traditional wet markets by modern buildings with the standards of department stores where no living animals should be stored and sold. Although obvious, this is very difficult to implement and must be accompanied by strong political actions. It is essential to ban the use of protected species and to enforce this prohibition but also to offer alternatives: (1) traditional pharmacopeia shops must be under government control; (2) the products sold must be validated by an official Academy; (3) full traceability, quality, and safety controls must be mandatory and internationally controlled, and (4) of upmost importance, products must be subsidized to ensure highly competitive prices to prevent a black market. In addition, customers should not be in contact with food, which should be provided by properly equipped staff members. It will also be necessary to ensure that farm animals do not end up in contact with wildlife.

## Beyond COVID-19

Although we specifically address COVID-19 and further Sarbecovirus pandemics here, examples and recommendations go far beyond. A future Sarbecovirus emergence will certainly involve East Asia due to the specific ecology of this group of viruses and their bat hosts. However, other epidemics can be triggered elsewhere. Middle East Respiratory Syndrome (MERS) is caused by a highly pathogenic Merbecovirus, a different Betacoronavirus, with a death rate of 34.7% (25, 26). MERS emerged in the Arabic Peninsula, with dromedaries as intermediate hosts, but the origin is found in African dromedaries and bats (27, 28). Countries from the Horn of Africa are breeding and trading dromedaries in the Arabic peninsula (27). The trade of live camels provided the amplification loop needed for the emergence of the disease. The emergence of a pandemic could happen in Africa through another intermediate host if an accidental amplification loop occurs. Another example comes from a different kind of virus: the mosquito-borne arboviruses. Their expansion is a consequence of the global economy and international trade, which led to the establishment of competent mosquito vectors, i.e., *Aedes albopictus* and *Aedes aegypti*, in many countries worldwide, including Europe. Large epidemics can then be triggered by international human mobility. This favored the emergence of Dengue, Chikungunya, and Zika in regions of the world where these viruses were absent, and it could happen again in the future.

## The Threat is Global, But the Answer is Local

Other pandemics will happen. It is just a matter of probability and time. Currently, the risk of emergence is mostly coming from coronaviruses, arboviruses, and influenza viruses. Influenza is given considerable scrutiny, and vaccines are available, making coronaviruses and arboviruses the main threat. We should, whenever possible, address the threat before it is recognized as a disease. Instead, all official actions taken today are post-event reactions, only aiming at reducing the progression of the disease. At this stage, the infectious agent has already spread, mostly during the incubation phase, and it is too late to efficiently stop it, whereas irreversible damage is being inflicted on people, society, and economies. A country is nothing else than the sum of her communities, and while rules must be international with a national liability for enforcement, the implementation must be delocalized to the community level. Different diseases will require different preventive actions, but these actions will all be efficient and easy to implement if they are managed at the community level. Whether it is the recommendations mentioned above for coronaviruses or control of mosquitoes, the community is the place where monitoring and preventive actions can be implemented quickly, efficiently, and at the lowest cost. International institutions and foundations can support low-income countries to implement this first range of local preventive measures. Indeed, the implementation of such recommendations will be far less expensive than the current cost of containment and devastation to the economy, which is counted in thousands of billions. Preparedness and education is therefore the utmost priority. It should be an international endeavor, and it is vital for governments to anticipate and prepare to stop the next emerging pandemic at the origin instead of just reacting and causing long-lived destruction to our society and economy, as we do today.

## Author Contributions

RF, ML, JS-C, and CD jointly proposed the idea and designed the study, performed the literature search, and collected data for a specific section of the manuscript each. All four authors participated in the writing and correction of the manuscript. RF and CD made the figure.

## Conflict of Interest

The authors declare that the research was conducted in the absence of any commercial or financial relationships that could be construed as a potential conflict of interest.
